# Supplementary material for: Translational Aspects in Metaplastic Breast Carcinoma
Source: Cancers (Basel). 2024 Apr 7;16(7):1433. doi: 10.3390/cancers16071433 (PMC11011105; doi:10.3390/cancers16071433)
Supplement: Supplementary file 1 [file cancers-16-01433-s001.zip › cancers-2885905-Supplementary files_corrected.pptx]

## Slide 1
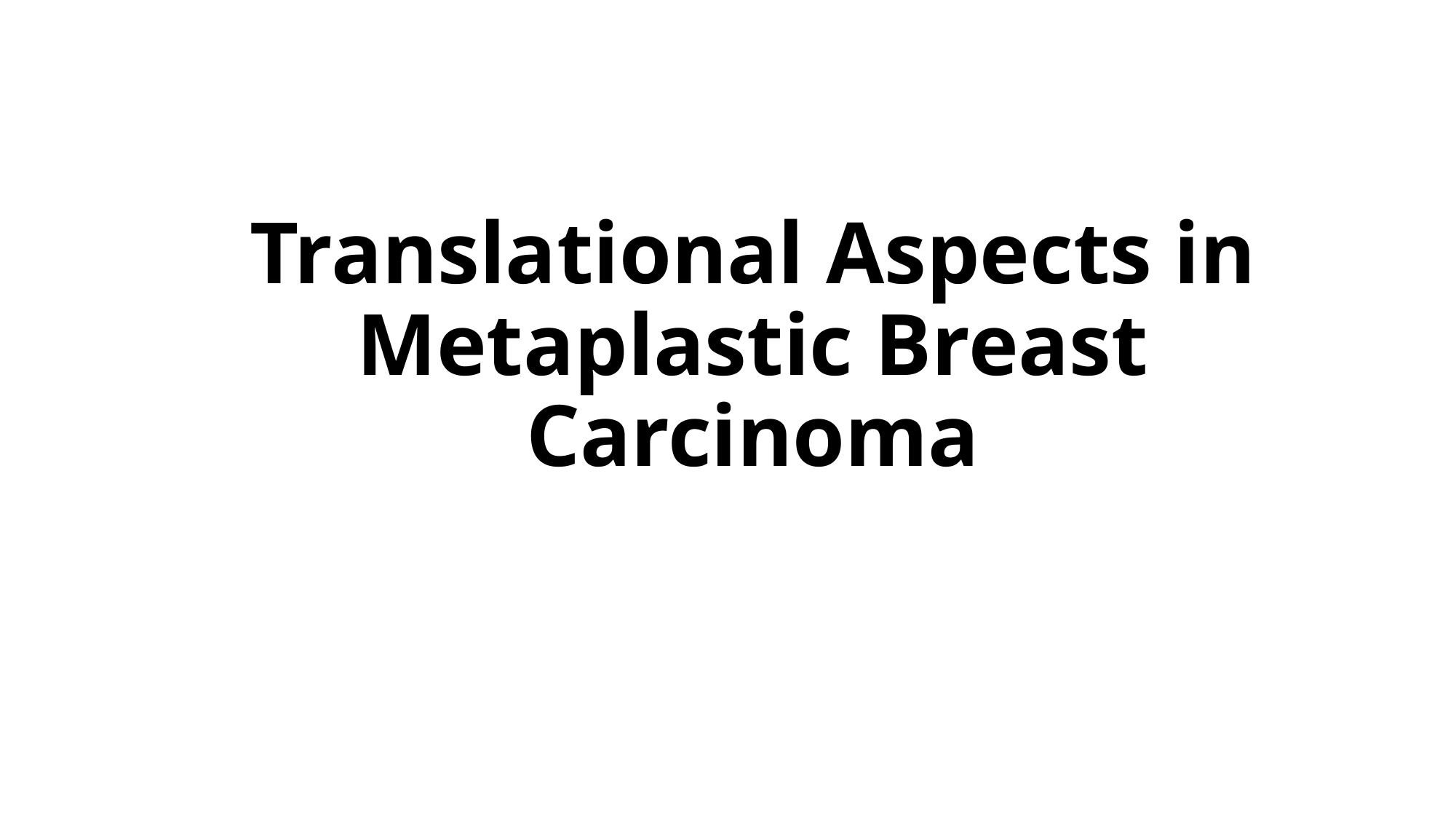

# Translational Aspects in Metaplastic Breast Carcinoma

## Slide 2
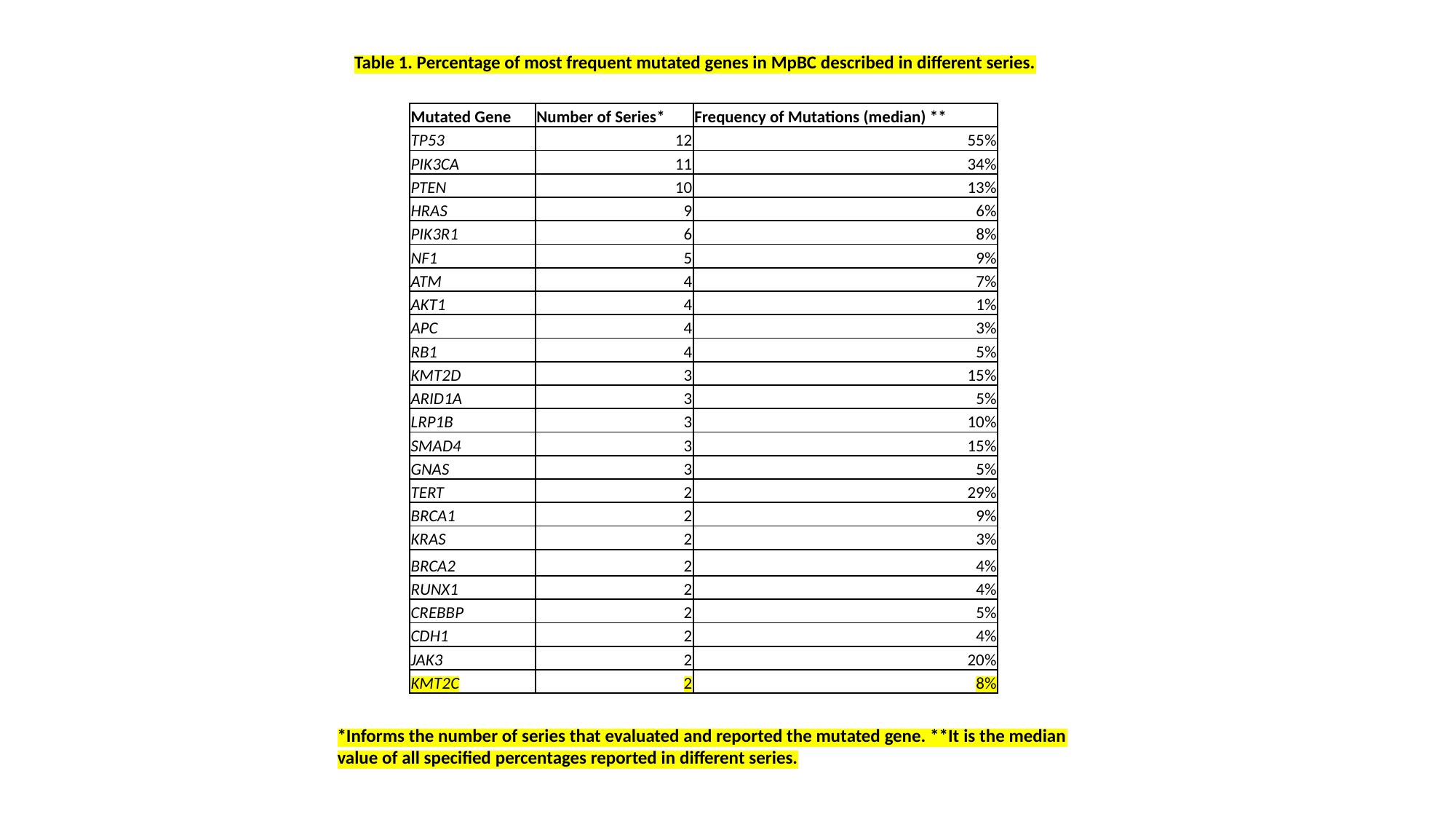

Table 1. Percentage of most frequent mutated genes in MpBC described in different series.
| Mutated Gene | Number of Series\* | Frequency of Mutations (median) \*\* |
| --- | --- | --- |
| TP53 | 12 | 55% |
| PIK3CA | 11 | 34% |
| PTEN | 10 | 13% |
| HRAS | 9 | 6% |
| PIK3R1 | 6 | 8% |
| NF1 | 5 | 9% |
| ATM | 4 | 7% |
| AKT1 | 4 | 1% |
| APC | 4 | 3% |
| RB1 | 4 | 5% |
| KMT2D | 3 | 15% |
| ARID1A | 3 | 5% |
| LRP1B | 3 | 10% |
| SMAD4 | 3 | 15% |
| GNAS | 3 | 5% |
| TERT | 2 | 29% |
| BRCA1 | 2 | 9% |
| KRAS | 2 | 3% |
| BRCA2 | 2 | 4% |
| RUNX1 | 2 | 4% |
| CREBBP | 2 | 5% |
| CDH1 | 2 | 4% |
| JAK3 | 2 | 20% |
| KMT2C | 2 | 8% |
*Informs the number of series that evaluated and reported the mutated gene. **It is the median value of all specified percentages reported in different series.

## Slide 3
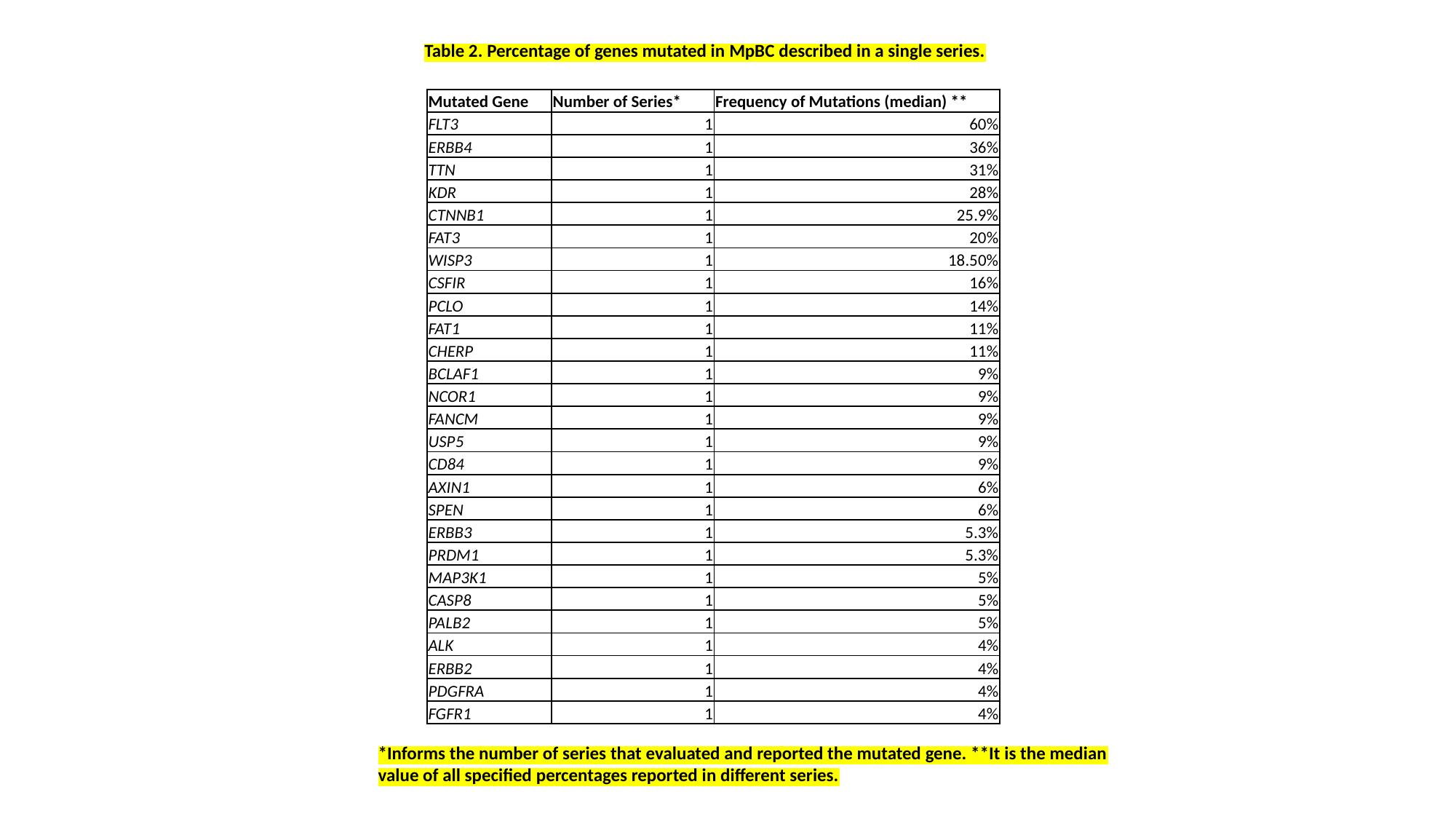

Table 2. Percentage of genes mutated in MpBC described in a single series.
| Mutated Gene | Number of Series\* | Frequency of Mutations (median) \*\* |
| --- | --- | --- |
| FLT3 | 1 | 60% |
| ERBB4 | 1 | 36% |
| TTN | 1 | 31% |
| KDR | 1 | 28% |
| CTNNB1 | 1 | 25.9% |
| FAT3 | 1 | 20% |
| WISP3 | 1 | 18.50% |
| CSFIR | 1 | 16% |
| PCLO | 1 | 14% |
| FAT1 | 1 | 11% |
| CHERP | 1 | 11% |
| BCLAF1 | 1 | 9% |
| NCOR1 | 1 | 9% |
| FANCM | 1 | 9% |
| USP5 | 1 | 9% |
| CD84 | 1 | 9% |
| AXIN1 | 1 | 6% |
| SPEN | 1 | 6% |
| ERBB3 | 1 | 5.3% |
| PRDM1 | 1 | 5.3% |
| MAP3K1 | 1 | 5% |
| CASP8 | 1 | 5% |
| PALB2 | 1 | 5% |
| ALK | 1 | 4% |
| ERBB2 | 1 | 4% |
| PDGFRA | 1 | 4% |
| FGFR1 | 1 | 4% |
*Informs the number of series that evaluated and reported the mutated gene. **It is the median value of all specified percentages reported in different series.

## Slide 4
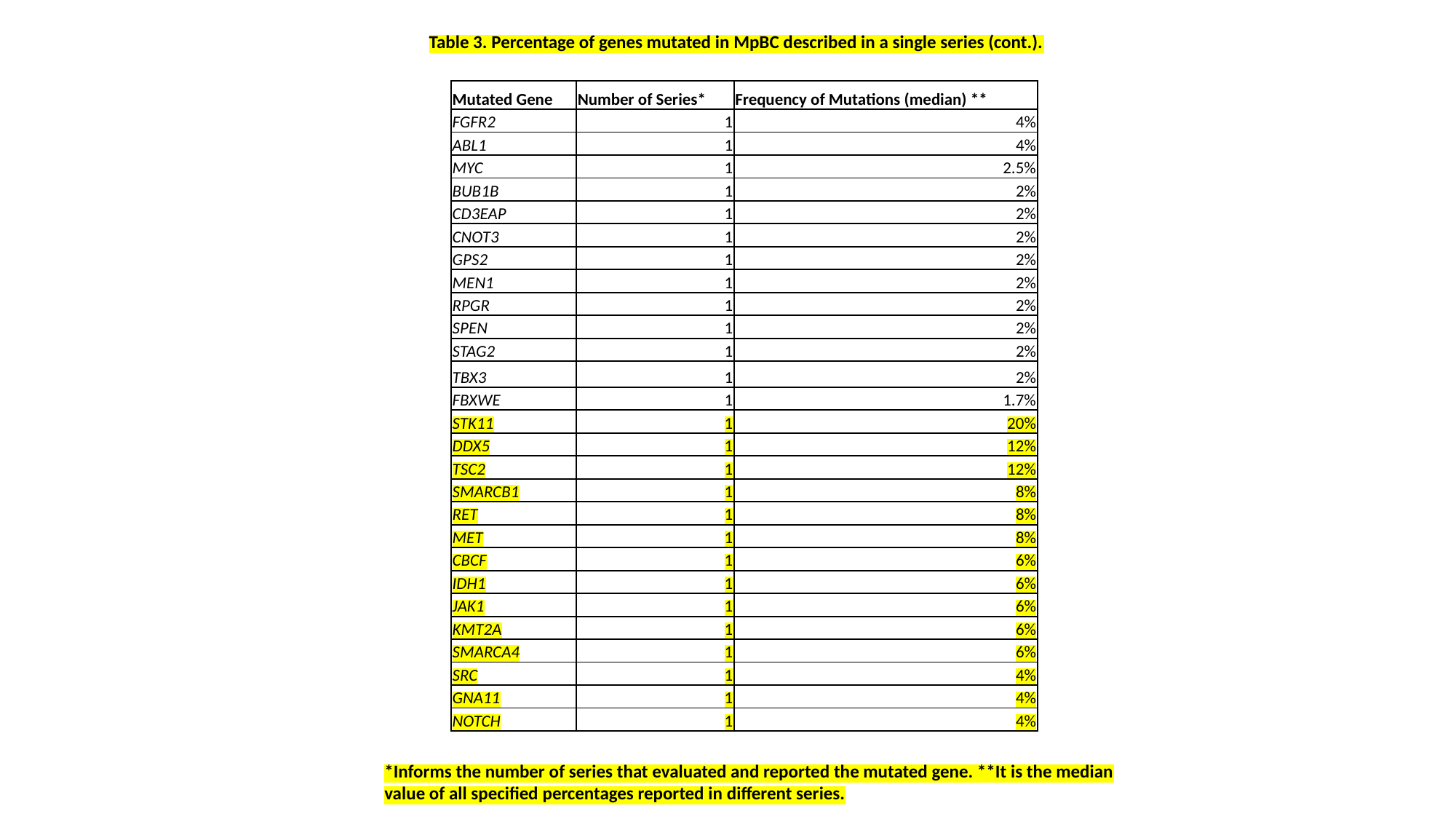

Table 3. Percentage of genes mutated in MpBC described in a single series (cont.).
| Mutated Gene | Number of Series\* | Frequency of Mutations (median) \*\* |
| --- | --- | --- |
| FGFR2 | 1 | 4% |
| ABL1 | 1 | 4% |
| MYC | 1 | 2.5% |
| BUB1B | 1 | 2% |
| CD3EAP | 1 | 2% |
| CNOT3 | 1 | 2% |
| GPS2 | 1 | 2% |
| MEN1 | 1 | 2% |
| RPGR | 1 | 2% |
| SPEN | 1 | 2% |
| STAG2 | 1 | 2% |
| TBX3 | 1 | 2% |
| FBXWE | 1 | 1.7% |
| STK11 | 1 | 20% |
| DDX5 | 1 | 12% |
| TSC2 | 1 | 12% |
| SMARCB1 | 1 | 8% |
| RET | 1 | 8% |
| MET | 1 | 8% |
| CBCF | 1 | 6% |
| IDH1 | 1 | 6% |
| JAK1 | 1 | 6% |
| KMT2A | 1 | 6% |
| SMARCA4 | 1 | 6% |
| SRC | 1 | 4% |
| GNA11 | 1 | 4% |
| NOTCH | 1 | 4% |
*Informs the number of series that evaluated and reported the mutated gene. **It is the median value of all specified percentages reported in different series.

## Slide 5
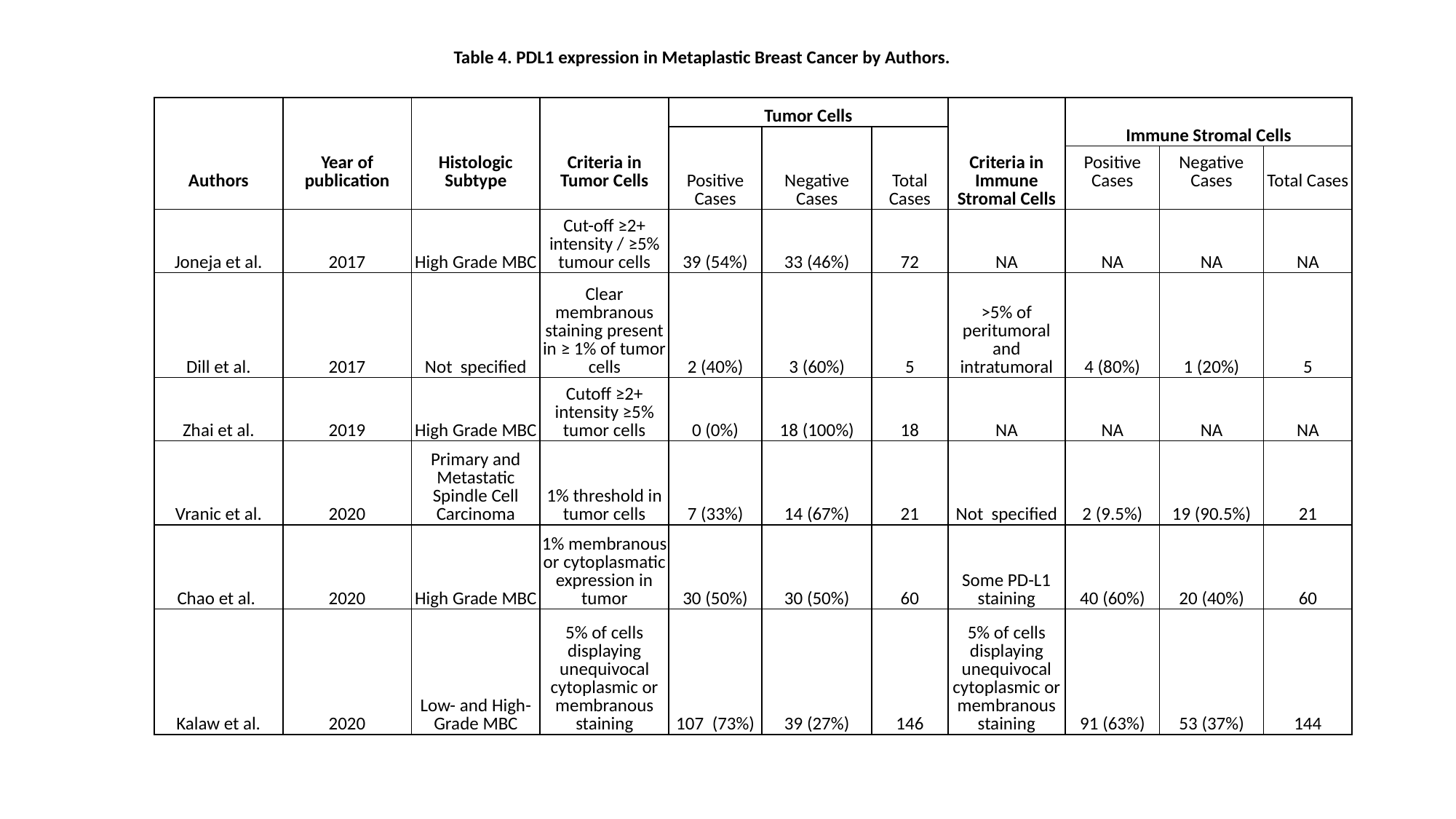

Table 4. PDL1 expression in Metaplastic Breast Cancer by Authors.
| Authors | Year of publication | Histologic Subtype | Criteria in Tumor Cells | Tumor Cells | | | Criteria in Immune Stromal Cells | Immune Stromal Cells | | |
| --- | --- | --- | --- | --- | --- | --- | --- | --- | --- | --- |
| | | | | Positive Cases | Negative Cases | Total Cases | | | | |
| | | | | Positive Cases | Negative Cases | Total Cases | | Positive Cases | Negative Cases | Total Cases |
| Joneja et al. | 2017 | High Grade MBC | Cut-off ≥2+ intensity / ≥5% tumour cells | 39 (54%) | 33 (46%) | 72 | NA | NA | NA | NA |
| Dill et al. | 2017 | Not specified | Clear membranous staining present in ≥ 1% of tumor cells | 2 (40%) | 3 (60%) | 5 | >5% of peritumoral and intratumoral | 4 (80%) | 1 (20%) | 5 |
| Zhai et al. | 2019 | High Grade MBC | Cutoff ≥2+ intensity ≥5% tumor cells | 0 (0%) | 18 (100%) | 18 | NA | NA | NA | NA |
| Vranic et al. | 2020 | Primary and Metastatic Spindle Cell Carcinoma | 1% threshold in tumor cells | 7 (33%) | 14 (67%) | 21 | Not specified | 2 (9.5%) | 19 (90.5%) | 21 |
| Chao et al. | 2020 | High Grade MBC | 1% membranous or cytoplasmatic expression in tumor | 30 (50%) | 30 (50%) | 60 | Some PD-L1 staining | 40 (60%) | 20 (40%) | 60 |
| Kalaw et al. | 2020 | Low- and High-Grade MBC | 5% of cells displaying unequivocal cytoplasmic or membranous staining | 107 (73%) | 39 (27%) | 146 | 5% of cells displaying unequivocal cytoplasmic or membranous staining | 91 (63%) | 53 (37%) | 144 |

## Slide 6
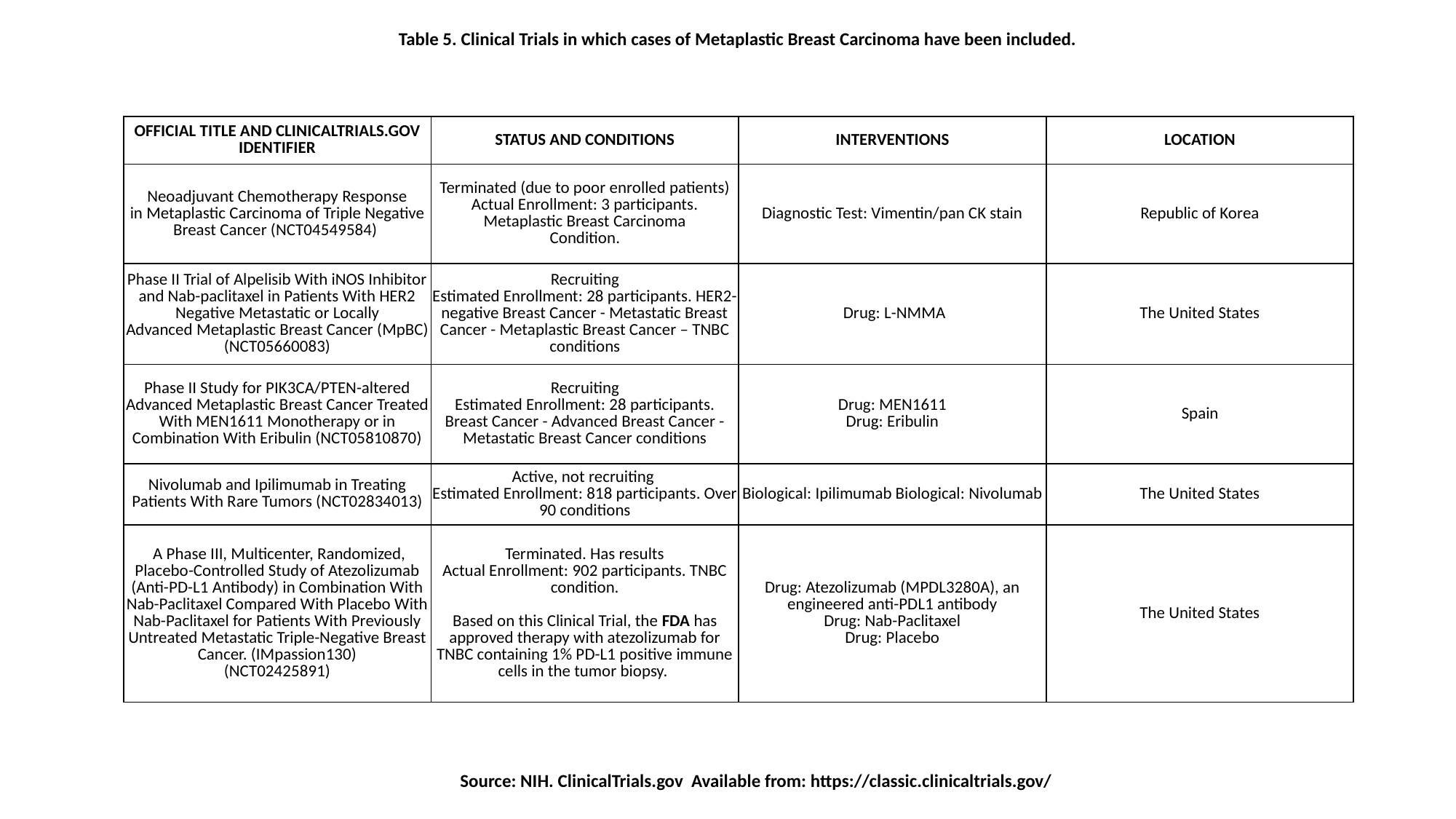

Table 5. Clinical Trials in which cases of Metaplastic Breast Carcinoma have been included.
| OFFICIAL TITLE AND CLINICALTRIALS.GOV IDENTIFIER | STATUS AND CONDITIONS | INTERVENTIONS | LOCATION |
| --- | --- | --- | --- |
| Neoadjuvant Chemotherapy Response in Metaplastic Carcinoma of Triple Negative Breast Cancer (NCT04549584) | Terminated (due to poor enrolled patients) Actual Enrollment: 3 participants. Metaplastic Breast Carcinoma Condition. | Diagnostic Test: Vimentin/pan CK stain | Republic of Korea |
| Phase II Trial of Alpelisib With iNOS Inhibitor and Nab-paclitaxel in Patients With HER2 Negative Metastatic or Locally Advanced Metaplastic Breast Cancer (MpBC) (NCT05660083) | Recruiting Estimated Enrollment: 28 participants. HER2-negative Breast Cancer - Metastatic Breast Cancer - Metaplastic Breast Cancer – TNBC conditions | Drug: L-NMMA | The United States |
| Phase II Study for PIK3CA/PTEN-altered Advanced Metaplastic Breast Cancer Treated With MEN1611 Monotherapy or in Combination With Eribulin (NCT05810870) | Recruiting Estimated Enrollment: 28 participants. Breast Cancer - Advanced Breast Cancer - Metastatic Breast Cancer conditions | Drug: MEN1611 Drug: Eribulin | Spain |
| Nivolumab and Ipilimumab in Treating Patients With Rare Tumors (NCT02834013) | Active, not recruiting Estimated Enrollment: 818 participants. Over 90 conditions | Biological: Ipilimumab Biological: Nivolumab | The United States |
| A Phase III, Multicenter, Randomized, Placebo-Controlled Study of Atezolizumab (Anti-PD-L1 Antibody) in Combination With Nab-Paclitaxel Compared With Placebo With Nab-Paclitaxel for Patients With Previously Untreated Metastatic Triple-Negative Breast Cancer. (IMpassion130) (NCT02425891) | Terminated. Has results Actual Enrollment: 902 participants. TNBC condition. Based on this Clinical Trial, the FDA has approved therapy with atezolizumab for TNBC containing 1% PD-L1 positive immune cells in the tumor biopsy. | Drug: Atezolizumab (MPDL3280A), an engineered anti-PDL1 antibody Drug: Nab-Paclitaxel Drug: Placebo | The United States |
Source: NIH. ClinicalTrials.gov Available from: https://classic.clinicaltrials.gov/

## Slide 7
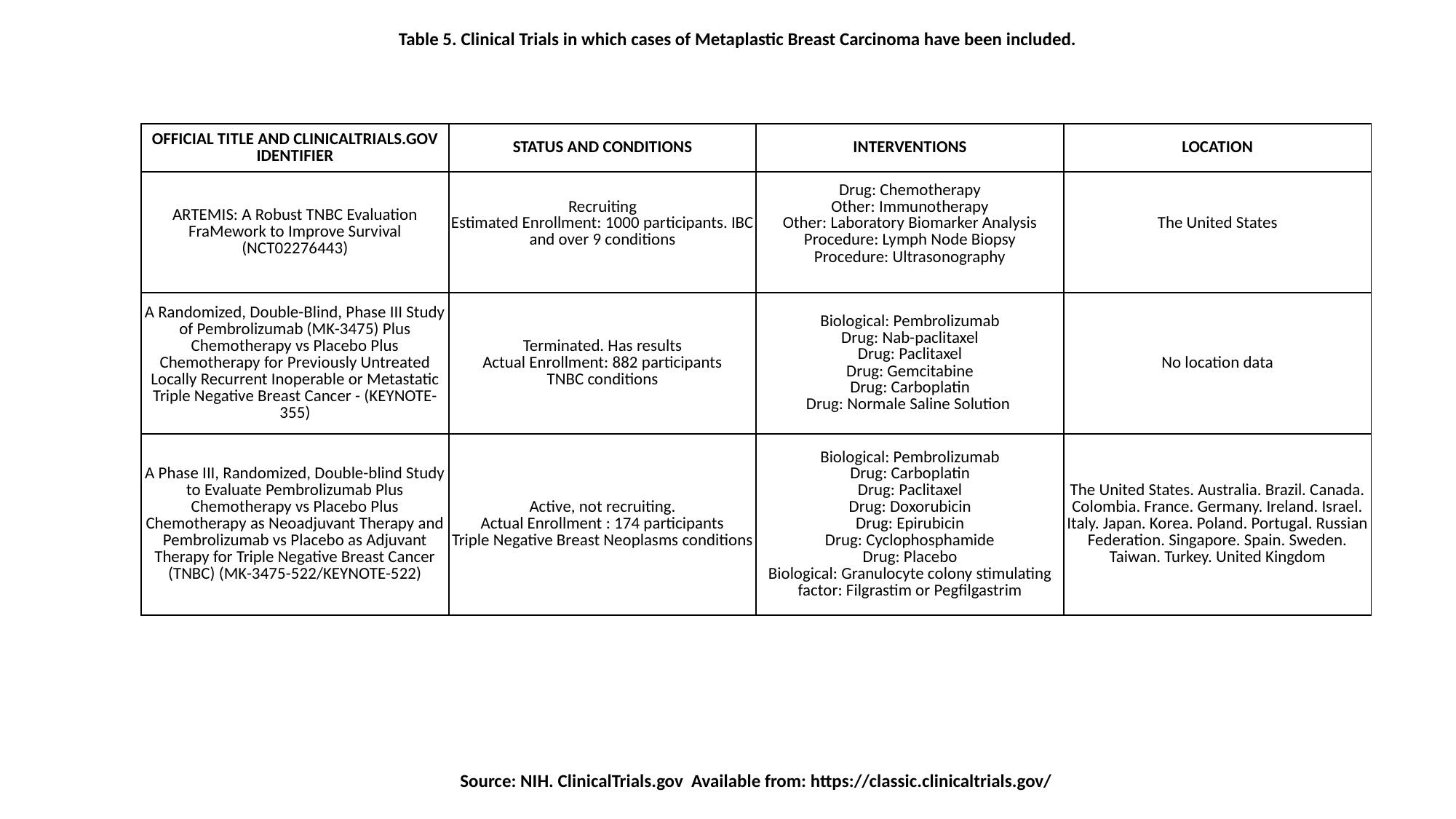

Table 5. Clinical Trials in which cases of Metaplastic Breast Carcinoma have been included.
| OFFICIAL TITLE AND CLINICALTRIALS.GOV IDENTIFIER | STATUS AND CONDITIONS | INTERVENTIONS | LOCATION |
| --- | --- | --- | --- |
| ARTEMIS: A Robust TNBC Evaluation FraMework to Improve Survival (NCT02276443) | Recruiting Estimated Enrollment: 1000 participants. IBC and over 9 conditions | Drug: Chemotherapy Other: Immunotherapy Other: Laboratory Biomarker Analysis Procedure: Lymph Node Biopsy Procedure: Ultrasonography | The United States |
| A Randomized, Double-Blind, Phase III Study of Pembrolizumab (MK-3475) Plus Chemotherapy vs Placebo Plus Chemotherapy for Previously Untreated Locally Recurrent Inoperable or Metastatic Triple Negative Breast Cancer - (KEYNOTE-355) | Terminated. Has results Actual Enrollment: 882 participants TNBC conditions | Biological: Pembrolizumab Drug: Nab-paclitaxel Drug: Paclitaxel Drug: Gemcitabine Drug: Carboplatin Drug: Normale Saline Solution | No location data |
| A Phase III, Randomized, Double-blind Study to Evaluate Pembrolizumab Plus Chemotherapy vs Placebo Plus Chemotherapy as Neoadjuvant Therapy and Pembrolizumab vs Placebo as Adjuvant Therapy for Triple Negative Breast Cancer (TNBC) (MK-3475-522/KEYNOTE-522) | Active, not recruiting. Actual Enrollment : 174 participants Triple Negative Breast Neoplasms conditions | Biological: Pembrolizumab Drug: Carboplatin Drug: Paclitaxel Drug: Doxorubicin Drug: Epirubicin Drug: Cyclophosphamide Drug: Placebo Biological: Granulocyte colony stimulating factor: Filgrastim or Pegfilgastrim | The United States. Australia. Brazil. Canada. Colombia. France. Germany. Ireland. Israel. Italy. Japan. Korea. Poland. Portugal. Russian Federation. Singapore. Spain. Sweden. Taiwan. Turkey. United Kingdom |
Source: NIH. ClinicalTrials.gov Available from: https://classic.clinicaltrials.gov/
